# Utilizing Microbial Electrochemical Methods to Enhance Lycopene Production in *Rhodopseudomonas palustris*

**DOI:** 10.3390/foods13233811

**Published:** 2024-11-26

**Authors:** Ningxin Huang, Zhengxiao Wang, Xiao Xiao, Te’er Gai, Dongyue Zhao, Lu Liu, Wei Wu

**Affiliations:** 1 College of Food Science and Engineering, Qingdao Agricultural University, Qingdao 266109, China; halo_huang1011@163.com (N.H.); wangzx1111@126.com (Z.W.); 17616196910@163.com (T.G.); aurora11236@163.com (D.Z.); snacksliu@163.com (L.L.); 2 Academy of Dongying Efficient Agricultural Technology and Industry on Saline and Alkaline Land in Collaboration with Qingdao Agricultural University, Dongying 257500, China; 3 Advanced Agri-Tech Institute, Qingdao Agricultural University, Qingdao 266109, China; 16678702919@163.com; 4 Qingdao Institute of Special Food, Qingdao Agricultural University, Qingdao 266109, China

**Keywords:** microbial electrosynthesis, *Rhodopseudomonas palustris*, lycopene production, mutant strains, gene analysis

## Abstract

Utilizing *Rhodopseudomonas palustris* (*R. pal*), this study constructed a dual-chamber microbial electrosynthesis system, based on microbial electrolysis cells, that was capable of producing lycopene. Cultivation within the electrosynthesis chamber yielded a lycopene concentration of 282.3722 mg/L when the optical density (OD) reached 0.6, which was four times greater than that produced by original strains. The mutant strain showed significantly higher levels of extracted riboflavin compared to the wild-type strain, and the riboflavin content of the mutant strain was 61.081 mg/L, which was more than 10 times that of the original strain. Furthermore, sequencing and analyses were performed on the mutant strains observed during the experiment. The results indicated differences in antibiotic resistance genes, carbohydrate metabolism-related genes, and the frequencies of functional genes between the mutant and original strains. The mutant strain displayed potential advantages in specific antibiotic resistance and carbohydrate degradation capabilities, likely attributable to its adaptation to electrogenic growth conditions. Moreover, the mutant strain demonstrated an enrichment of gene frequencies associated with transcriptional regulation, signal transduction, and amino acid metabolism, suggesting a complex genetic adaptation to electrogenic environments. This study presents a novel approach for the efficient and energy-conserving production of lycopene while also providing deeper insights into the genetic basis of electro-resistance genes.

## 1. Introduction

Lycopene, a natural pigment found in tomatoes and various other red fruits, has attracted considerable attention owing to its potent antioxidant properties and potential health benefits [1]. Lycopene is widely found in tomatoes, watermelon, red grapefruit, apricots, and other red- or pink-colored fruits and vegetables, such as red or pink oranges, and can also be synthesized by carotenoid-producing microorganism [2,3,4,5,6]. As a carotenoid, lycopene is a crucial component of human nutrition, demonstrating remarkable antioxidant activity that neutralizes harmful free radicals in the body, and it can only be supplied through a variety of forms of intake such as food or medication [2]. Furthermore, lycopene is associated with several health benefits, including a reduced risk of specific chronic diseases, such as cardiovascular disease and certain types of cancer [7], and can improve human immunity and regulate blood lipids [8]. Therefore, it is necessary to produce lycopene on a large scale.

At present, the production methods of lycopene mainly include natural extraction methods and microbial synthesis. Traditionally, lycopene has primarily been extracted from tomato fruits and processed into various commercial products [9]. However, there are several limitations and drawbacks to this traditional method of producing lycopene. First, the extraction process is usually labor-intensive, time-consuming, costly, and requires a lot of resources and energy [10,11]. Furthermore, reliance on the agricultural cultivation of tomatoes for lycopene extraction exacerbates environmental concerns, including land use, water consumption, and pesticide usage [12]. However, the production of lycopene from plants is costly and environmentally unfriendly [13]. Microbial synthesis method has the advantages of low production costs, high efficiency, and environmental friendliness. There are two types of microbial lycopene production; one is microorganisms that can naturally synthesize lycopene, such as *Blakesleatrispora* [14]. Studies have shown that the yield of lycopene produced by *B. trisporus* has been continuously improved, and the highest lycopene yield has been reported to be 3.4 g/L [15]. However, *B. trispora* is susceptible to degradation during passaging, resulting in unstable yields and low production efficiency [16]. On the other hand, recombinant strains can perform the heterologous de novo synthesis of lycopene, which are usually model microorganisms with mature molecular manipulation and clear genetic backgrounds, such as *Escherichia coli* and *Saccharomyces cerevisiae* [16]. However, the fermentation process of *E. coli* is susceptible to phage infection and poses a risk of endotoxin secretion; *S. cerevisiae* has a long fermentation cycle and high production costs. With the advancement of biotechnology, many methods such as metabolic engineering have been employed to optimize the efficiency of lycopene synthesis [6].

In recent years, there have been more reports on the microbial biosynthesis of lycopene; microbial electrosynthesis has emerged as a promising alternative for the sustainable and efficient production of valuable compounds, including lycopene [17,18,19]. Microbial electrochemical processes are carried out in bioelectrochemical systems, an emerging sustainable technique for catalytic reduction or oxidation reactions, in which bacteria interact with electrodes and exchange electrons between them [20,21,22,23]. Microbial electrosynthesis presents several advantages over traditional chemical and biological production methods, including higher product yields, reduced resource inputs, and a minimal environmental impact [24]. By leveraging the unique metabolic pathways of microorganisms, microbial electrosynthesis represents a promising avenue for the cost-effective and environmentally friendly production of lycopene and other valuable bioactive compounds [25].

This innovative biotechnological process harnesses the metabolic capabilities of microorganisms, such as *R. pal*, to bioconvert carbon dioxide or organic substrates into target compounds, utilizing electricity as an energy source [6,26]. *R. pal* is a purple photosynthetic bacterium with chlorophyll and carotenoids that is able to utilize light energy to synthesize organic matter and thus photosynthesize. This metabolic process primarily relies on the mechanism of purple bacterial photosynthesis [27,28]. The bacterium *R. pal* exhibits several advantageous characteristics. It is capable of anaerobic growth in the presence of light and is capable of photosynthesis, using light energy to synthesize organic matter. It has two photosynthetic mechanisms, one of which is photosynthesis by purple photosynthetic bacteria, which uses light energy to produce ATP and converts light energy into chemical energy through an electron transport chain [27,29,30,31]. The other is heterotrophic growth, in which the bacterium can grow heterotrophically in the absence of light, using organic matter as a carbon source [32]. *R. pal* has potential as a bacterium for biotechnological applications and is of great interest in the synthesis of lycopene. Compared to traditional production methods, microbial electrosynthesis technology is a green and environmentally friendly production method, which does not produce greenhouse gasses such as carbon dioxide, which is conducive to reducing environmental pollution and greenhouse gas emissions. Moreover, microbial electrosynthesis technology has demonstrated extensive application prospects in fields such as wastewater treatment, biosensors, and food processing and production [33]. It can also be utilized for the production of high-value-added products like hydrogen, organic acids, and biopolymers, featuring favorable economic benefits and environmental friendliness.

In this context, this study aims to investigate the feasibility of employing *R. pal* in a microbial electrosynthesis system for the efficient production of lycopene by elucidating the metabolic pathways and genetic mechanisms involved in lycopene biosynthesis, as well as analyzing mutant strains to enhance lycopene production. This research aims to advance our understanding of microbial electrosynthesis and its potential applications in sustainable biomanufacturing, providing new ideas and methods for the further development of microbial electrosynthesis technology and providing new options for efficient, green, and safe lycopene production methods.

## 2. Materials and Methods

### 2.1. Materials

The strain utilized in this experiment was a laboratory-prepared strain of *Rhodopseudomonas palustris* (RPLYC45). Chemicals, including 4-Amino-benzenesulfonic acid monosodium salt (CAS: 515-74-2), were procured from MACKLIN. Potassium phosphate monohydrate (CAS: 7778-77-0) and ammonium sulfate (CAS: 7783-20-2) were obtained from Tianjin Boditech Chemical Co., Ltd., Tianjin, China. Sodium thiosulfate pentahydrate (CAS: 10102-17-7) and disodium hydrogen phosphate dodecahydrate (CAS: 10039-32-4) were purchased from China National Pharmaceutical Group Chemical Reagent Co., Ltd., Shanghai, China. The experimental setup also included standard laboratory materials and reagents for microbial culture maintenance, DNA extraction, and sequencing. All chemicals and materials were used in accordance with established laboratory protocols to ensure experimental reproducibility and accuracy.

*R. pal* requires optimal growth conditions in PM medium, the preparation method of which is presented in Table 1. The method is as follows: Add ultrapure water to the prepared medium to a final volume of 1 L and adjust the pH to 6.8. Sterilize the solution using an autoclave, and subsequently store it at 30 °C for future use. In a laminar flow hood, incorporate yeast extract powder into the medium. Dissolve 1 g of yeast extract powder in the medium by extracting and filtering it through a 0.22 μm filter membrane using a syringe. Another PBS buffer is required, and the configuration method is shown in Table 2. Thoroughly stir the solution to ensure complete dissolution. Adjust the pH to 7.4 using concentrated HCl, and then add ultrapure water to reach a total volume of 1000 mL.

### 2.2. Activation of R. pal

Due to the anaerobic requirement for the cultivation of *R. pal*, nitrogen gas was employed to evacuate the culture medium, thereby creating an anaerobic sealed environment. Initially, a 15 cm needle was inserted into the bottom of the culture medium and de-gassed for 20 min. Subsequently, the bottle neck was rapidly sealed with a blue rubber stopper, which was pierced with a needle to puncture the stopper, and the needle was inserted deeply into the liquid culture medium. Two additional 1 mL needles were inserted into the blue rubber stopper to prevent excessive pressure within the bottle and were de-gassed for an additional 20 min. Afterward, the three needles were removed, and the bottle neck was sealed with a bottle pressurizer to establish an anaerobic environment.

Within a laminar flow hood, 1 mL of the original *R. pal* culture was aspirated using a disposable syringe and injected into 100 mL of the culture medium. The mixture was then incubated in a temperature-controlled illuminated incubator at 30 °C until the culture medium exhibited a red coloration. Following thorough mixing, an inoculum comprising 2% of the liquid content of the experimental culture medium was transferred, and the aforementioned steps were repeated.

### 2.3. Initial Start-Up of the Electrochemical System

Preparation of carbon felt, an anion exchange membrane, titanium wire, and dual-chamber electrochemical cells was conducted. Three pieces of carbon felt were cut into 3 × 3 cm squares, each designated for use as the counter electrode, anode, and cathode, with a salt bridge incorporated into each. Additionally, a Ag/AgCl reference electrode was utilized for calibration, serving as a reference to complete the initial setup. Sodium bicarbonate (NaHCO_3_) was added to the culture medium in both the cathode and anode chambers to function as an electron acceptor. Based on the volume of the PM medium in the cathode and anode chambers, 4.1005 g of sodium bicarbonate (NaHCO_3_) was added per liter of PM medium.

#### 2.3.1. Carbon Felt Electrode Treatment

Initially, the carbon felt was rinsed with ultrapure water, immersed in acetone, and subjected to 30 min of ultrasonic treatment to eliminate organic solvents. This step was repeated twice, followed by the soaking of the carbon felt in acetone for 24 h. Subsequently, the carbon felt was placed in 1 mol/L HCl and subjected to 30 min of ultrasonic treatment. This step was repeated twice, allowing the carbon felt to soak for 24 h each time to facilitate the removal of metal ions. Finally, the carbon felt was subjected to 30 min of ultrasonic treatment using ultrapure water. This step was repeated twice, permitting the carbon felt to soak for 24 h each time. Following rinsing with ultrapure water, the carbon felt was placed in an oven for drying.

#### 2.3.2. Acid Tank Cleaning of the Reactor

A diluted hydrochloric acid solution was prepared by mixing concentrated HCl with ultrapure water at a ratio of 1:15. The dual-chamber electrochemical cell was immersed in the diluted HCl solution for two days to facilitate the removal of metal ions, thereby ensuring the accuracy of subsequent experiments.

#### 2.3.3. Electrochemical Conditions

The current intensity of this experiment ranged from 1 to 2 A. Cyclic voltammetry was used for detection, the lowest potential was set to −1.5 V, and the highest potential was set to 1.5 V. The sensitivity of the electrochemical workstation was also set to 1.0 × e^−5^ A/V.

### 2.4. Characterization of R. pal

#### 2.4.1. Comparison of Growth OD_660_

*R. pal* was observed at different electrochemical cultivation times using Fourier transform infrared spectroscopy (FT-IR, Thermo, Waltham, MA, USA). The growth conditions of the cathode and anode in the dual-chamber electrolysis cell were compared at 660 nm, along with the original and mutant strains under electrochemical cultivation.

#### 2.4.2. Fourier Transform Infrared Spectroscopy (FT-IR)

*R. pal* was observed at different electrochemical cultivation times using FT-IR (Nicolet iS 10, Thermo, Waltham, MA, USA). The scanning range was 0–4000 cm^−1^, with a total of 10 scans. The scanning speed was up to 405 spectra per second, with a peak-to-peak noise level below 1.24 × 10^−5^ AU (using a DTGS detector and KBr window). The strains that underwent electrochemical incubation for 3 d, 6 d, 9 d, and 12 d were taken, cleaned, and fixed on sodium chloride wafers for FT-IR spectroscopy. Data processing followed the ASTM E1421 standards [34], using the software (OMNIC, https://www.thermofisher.cn/order/catalog/product/INQSOF018, accessed on 24 November 2024) provided with the instrument.

#### 2.4.3. Cyclic Voltammetry and Potentiometric Titration

*R. pal* at various OD_660_ levels was subjected to cyclic voltammetry using an electrochemical workstation (CHI760E, Chenhua, Shanghai, China). Additionally, the electrolyte was titrated with an automatic potentiometric titrator (Mettler Toledo, Greifensee, Switzerland) to assess electron transfer capabilities.

#### 2.4.4. DNA Gel Electrophoresis and Liquid-Phase Determination of Riboflavin Content

DNA gel electrophoresis was utilized to quantify the total DNA content of both the original and mutant strains of *R. pal* at various electrochemical cultivation times. The ratio of agarose gel was 1 (i.e., 0.6 g of agarose powder to 60 mL of 1× TAE buffer, with 2 μL of gel-red dye added). The electrophoresis conditions were as follows: the voltage was 10 V, and the electrophoresis time was 40 min. Subsequently, riboflavin was extracted from both the original and mutant strains, and its content was quantified using high-performance liquid chromatography (HPLC, U1timate3000, Thermo Fisher, Shanghai, China) for comparative analysis. The cultured original and mutant strains of *Pseudomonas aeruginosa* were collected and centrifuged for washing, which was repeated twice. The collected original and mutant strains were soaked in methanol for 15 min and the cell walls were broken using ultrasound to release intracellular riboflavin. Chloroform was used to mix with the crushed cells to dissolve the riboflavin in the organic phase, and the organic phase containing riboflavin from the original strain and the mutant strain was isolated. For the determination of riboflavin, a C18 column was used, and the mobile phase was set as acetonitrile–formic acid = 7:3 at a flow rate of 1.2 mL/min. The detection wavelength was set at 254 nm, and the temperature was maintained at 30 °C.

### 2.5. Production of Lycopene

The samples were centrifuged (v = 5500 r/min; t = 15 min, 4 °C) and the cell precipitates were collected from 1 mL each of the cultured *Pseudomonas aeruginosa* mutants. The cell precipitates were dried under vacuum, added into a mixture of methanol and hexane (the ratio of methanol to hexane was 1:1), and broken by ultrasonication (t = 20 min); then, the samples were centrifuged (v = 15,000 r/min; t = 20 min, 4 °C), and the supernatant (i.e., lycopene tissue solution) was collected for subsequent experimental measurements. The lycopene content was quantified using high-performance liquid chromatography (HPLC) with 500 μL of a lycopene standard solution and 500 μL of *R. pal* bog tissue extract. The chromatography was performed on a C18 column with mobile phase consisting of acetonitrile, methanol, and trichloromethane (the ratio by volume of these three substances was 42.5:42.5:15) at a flow rate of 1 mL/min, with an injection volume of 20 μL and a detection wavelength of 473 nm. For each group of treatments, five replicates were set up, and the sample size for each test was 1 mL of sap from each replicate.

### 2.6. Genomics and Pathway Analysis of Mutant Strains of R. pal

Firstly, suspensions of well-grown and healthy swamp-red pseudomonas from the original strain and mutant strain at logarithmic phase were selected and centrifuged at 5500 rpm for 15 min to obtain cell pellets, and this was followed by discarding the culture medium. An appropriate volume of 1× PBS was added to resuspend the cell pellets and then centrifuged at 200 rpm for 5 min. The PBS washing step was repeated twice. The resulting cell pellets were resuspended in 200 μL of buffer solution to completely suspend the cells. For Gram-positive bacteria with tougher cell walls, this step could be omitted, and, instead, the cells could be treated with lysozyme solution for cell wall disruption. This method involved adding 110 μL of buffer solution (20 mM Tris, pH 8.0; 2 mM Na2-EDTA; 1.2% Triton) and 70 μL of lysozyme solution, followed by incubation at 37 °C for at least 30 min. To remove RNA, 4 μL of RNase A (100 mg/mL) solution could be added, followed by brief vortexing for 15 s and incubation at room temperature for 5 min. Next, 20 μL of Proteinase K solution was added to the tube and mixed well, and then 220 μL of GB buffer solution was added. The mixture was vortexed for 15 s and incubated at 70 °C for 10 min until the solution became clear. After a brief centrifugation to remove water droplets from the inner wall of the tube cap, 220 μL of anhydrous ethanol was added, thoroughly mixed by vortexing for 15 s, and then briefly centrifuged to remove water droplets from the inner wall of the tube cap. The resulting solution and precipitate were transferred to an adsorption column (CB3) and centrifuged at 12,000 rpm for 30 s. The waste liquid was discarded, and the adsorption column (CB3) was placed in a collection tube. Subsequently, 500 μL of GD buffer solution was added to the adsorption column (CB3) and centrifuged at 12,000 rpm for 30 s. The waste liquid was discarded, and the adsorption column (CB3) was placed in a collection tube. Finally, 600 μL of PW wash solution was added to the adsorption column (CB3) and centrifuged at 12,000 rpm for 30 s. The waste liquid was discarded, and the adsorption column (CB3) was placed in a collection tube, thereby achieving successful DNA extraction. The extracted DNA was subsequently utilized for genome assembly using next-generation sequencing technology. An Illumina NovaSeq 6000 library (300–500 bp) was constructed, and the acquired sequencing data were subjected to quality control and bioinformatics analysis to complete the draft genome of the strain.

## 3. Results and Discussion

### 3.1. Construction of a Dual-Chamber Microbial Electrolysis Cell (MEC)

**Figure 1:**
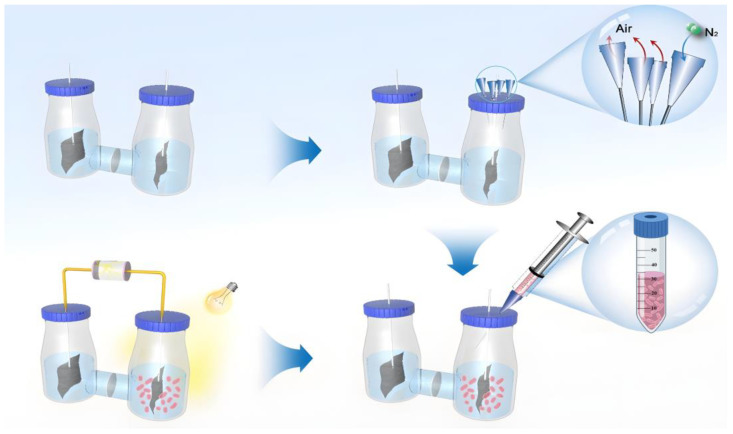
Dual-chamber MEC construction method.

The method for the construction of a dual-chamber microbial electrolysis cell is as follows: Bind the treated carbon felt electrodes onto two titanium wires, ensuring that they are placed separately into the dual-chamber electrochemical cell. Introduce 400 mL of PM medium into each chamber of the electrochemical cell. Insert anion exchange membranes between the chambers to ensure proper sealing, and connect the other ends of the titanium wires to the respective positive and negative poles of the battery. Subsequently, degas the cathode and anode separately with nitrogen gas according to the aforementioned procedure to establish anaerobic conditions, as illustrated in Figure 1.

The method for electrochemical cultivation is as follows: Initiate the centrifuge and pre-cool it to 4 °C. Once the *R. pal* culture reaches an OD_660_ of approximately 0.4, centrifuge it at 5500 rpm for 15 min to collect 50 mL of cells. Subsequently, centrifuge the cells (without an electron donor) to wash them, and repeat this step once. Resuspend the collected cells and introduce them into the dual-chamber electrochemical cell. Insert the assembled dual-chamber electrochemical system into the illuminated incubator, activate the power, and commence the electrochemical cultivation. It is noteworthy that the volume of culture medium in each reactor is 400 mL, with an inoculation of 20 mL of *R. pal* (adding 500 μL of cell suspension for every 10 mL), resulting in a final concentration of the electron acceptor, NaHCO_3_, at 20 mM.

### 3.2. Electrochemical Cultivation and Growth Conditions of R. pal

**Figure 2:**
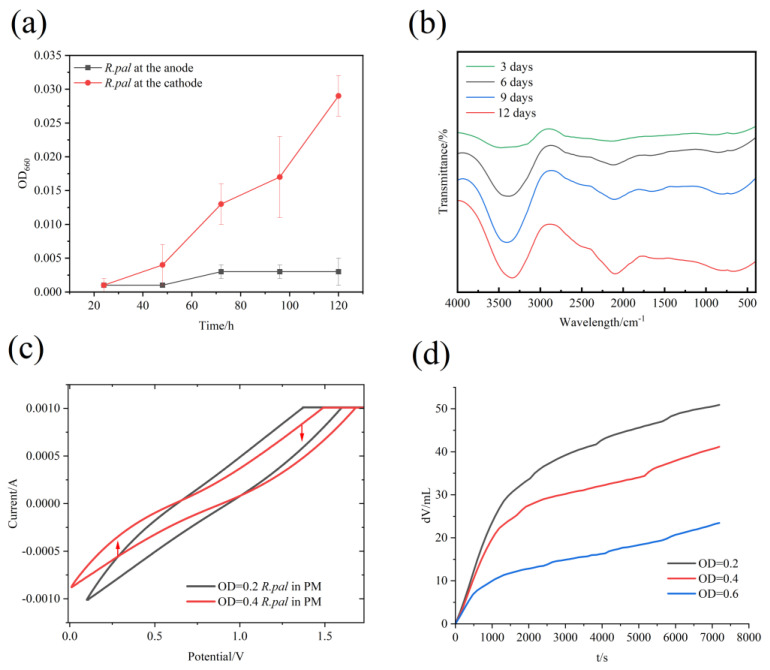
Characteristics of electrochemical culture of *R. pal*. (**a**) Comparison of growth of positive and negative strains of MES in double ponds. (**b**) Infrared spectrum scanning of strains under different culture days. (**c**) Infrared spectrum scanning of strains under different culture days. (**d**) Comparison of voltametric cyclic curves with OD_660_ = 0.2 and 0.4.

To investigate potential differences in the growth trends of *R. pal* at the cathode and anode of the dual-chamber MEC, we employed the cultivation method previously described. We added 400 mL of PM medium, devoid of sodium thiosulfate, to both the cathode and anode chambers of the dual-chamber cell. Then, 20 mL of activated *R. pal* with OD_660_ = 0.3 was added separately to the cathode and anode chambers. Following the application of electric current, electrochemical cultivation was performed. After five days of cultivation, the OD_660_ values at the cathode and anode were observed and compared, with the results presented in Figure 2a.

The experimental results indicate that the *R. pal* displayed a favorable growth trend at the cathode. After 120 h of cultivation, a distinct difference between the cathode and anode was visually evident. At the cathode, the OD_660_ of the *R. pal* reached 0.29 and continued to rise. In contrast, under the same electrochemical cultivation conditions, the *R. pal* at the anode displayed no growth trend, and its OD_660_ remained at baseline levels. This result suggests that, owing to the influence of the anion exchange membrane, electrons were effectively enriched at the cathode, facilitating the *R. pal’s* receipt of electrons from an external power source for growth. This finding illustrates an alternative method for supplying electrons, substituting organic compounds such as sodium thiosulfate.

Figure 2b presents the infrared spectroscopy scans of strains cultured electrochemically for 3, 6, 9, and 12 days. The results indicate that with increasing culture time, the peak area of the alkyne characteristic peak (2115 cm^−1^) increased, which may be associated with the enhanced photosensitivity of the photosynthetic bacteria. *R. pal*, a bacterium that is both photoreceptive and electro tolerant, exhibits enhanced photoreceptivity under electrochemical conditions. Due to its pigments, *R. pal* is capable of absorbing infrared wavelengths, allowing it to absorb more light energy and convert it into chemical energy for growth and metabolic activities. The absorption and utilization of light by photosynthetic bacteria are typically key factors in their growth and metabolic activity. Therefore, the increase in the alkyne content may reflect the enhanced ability of the strains to absorb and utilize light energy for growth and metabolic activities as the culture time progresses. Additionally, the vibration of the -OH bond observed at 3436.5 cm^−1^ may be related to the presence of water molecules and the formation of hydrogen bonds within the bacterial cells. As culture time increases, the characteristic peak of this -OH bond vibration may become more pronounced, indicating an increase in water molecules or the enhanced formation of hydrogen bonds within the cells. This phenomenon may be associated with changes in water content during bacterial growth and metabolic processes. Therefore, considering the changes in both the alkyne characteristic peak and the -OH bond vibration characteristic peak, it can be inferred that the photosensitivity of the photosynthetic bacteria may increase with increasing culture time, accompanied by changes in the cellular water content and the extent of hydrogen bond formation.

In order to investigate the internal electron transfer of *R. pal* under electrochemical conditions, we conducted cyclic voltammetry (CV) measurements. Firstly, we placed the PM medium reactors in an acid bath for 48 h to remove metal ions from the reactors, ensuring the smooth CV curves of the PM medium during the measurements to guarantee the stability of the internal electron transfer. Subsequently, we cultivated the mutant strain of *R. pal* under electrochemical conditions until the OD_660_ reached 0.2 and 0.4, respectively, and performed CV measurements for both the original and mutant strains. The experimental results showed that when the OD_660_ of the strain was 0.2, the CV curve exhibited significant fluctuations, with prominent contractions around 0.5 V and 1.1 V during the CV measurements. This indicates that the addition of the original strain of *R. pal* at an OD_660_ of 0.2 significantly altered the internal electron transfer, demonstrating the utilization effect of *R. pal* under an applied electric current, as shown in Figure 2c.

Furthermore, comparing the CV curves at OD_660_ = 0.4 to those at OD_660_ = 0.2, we found that the degree of fluctuation was more pronounced, with more intense fluctuations compared to the original strain. This suggests that with the progress of electrochemical cultivation, *R. pal* exhibits enhanced electron utilization efficiency, demonstrating better electrochemical tolerance and electron utilization efficiency.

To investigate the consumption of the substrate by *R. pal* in the electrochemical synthesis cell, we used the method of electron titration to titrate KH_2_PO_4_ in the substrate through redox reactions. We cultivated the strains to different OD_660_ values (0.2, 0.4, and 0.6), extracted 100 mL of the supernatant, and performed titration. The purpose of this was to detect, under the action of the anion exchange membrane, the utilization of electrons by the mutant strain at different OD_660_ values and to verify whether it can replace traditional organic compounds such as sodium thiosulfate to provide electrons, as shown in Figure 2d.

According to the experimental results, the KH_2_PO_4_ content in the substrate gradually decreased as the optical density of the growth increased. This indicates that the strains exhibited good growth characteristics during growth and could effectively utilize electrons in the substrate under electrochemical conditions.

### 3.3. Analysis of Lycopene Production

A total of 1 mL of the mutant strain of *R. pal* cultured to an OD_660_ of 0.2, 0.4, and 0.6, respectively, was taken and balanced. During the process of sample extraction, disposable sterile syringes were used to avoid disturbing the anaerobic environment in the bottles, preparing for subsequent hydrogen detection. Subsequently, the samples were placed in a high-speed refrigerated centrifuge pre-cooled to 4 °C for centrifugation. The centrifugation conditions were set to 5500 rpm for 15 min to collect cell pellets. After collection, the cell pellets were subjected to vacuum drying.

**Figure 3:**
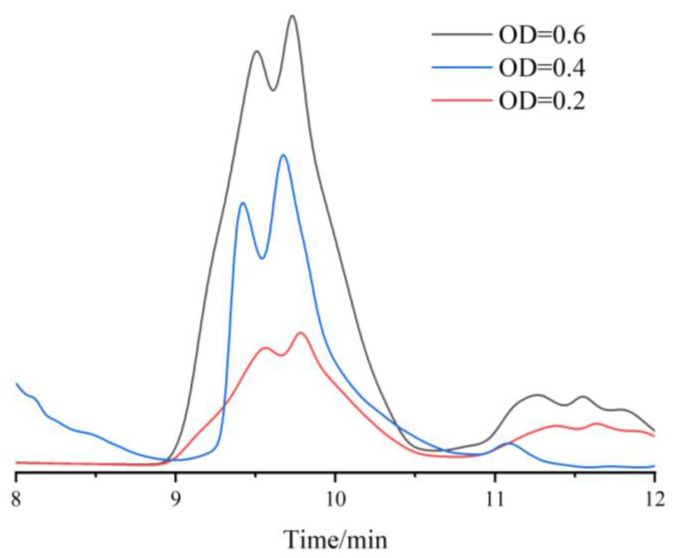
OD = 0.2, 0.4, and 0.6; liquid chromatogram of lycopene extracted by mutant strain was obtained.

Next, the vacuum-dried cell pellets were added to a mixture of methanol and n-hexane (in a ratio of 1:1) and subjected to ultrasonic disruption for 20 min. The samples were then centrifuged at 15,000 rpm for 20 min to collect 500 μL of supernatant, which was analyzed using HPLC, as shown in Figure 3.

According to the experimental results, the peak retention time of the lycopene standard in HPLC was approximately 9.8 min. Similarly, a distinct peak was observed at the same retention time in the crude extract of *R. pal*. This result indicates that the *R. pal* successfully synthesized lycopene using this experimental method. The peak areas corresponding to OD = 0.2, OD = 0.4, and OD = 0.6 were 88.0103 mAU, 196.9056 mAU, and 263.7035 mAU, respectively.

Based on the linear regression equation Y = 1.2076X − 77.2892, the lycopene content was calculated to be 136.8827 mg/L, 227.0576 mg/L, and 282.3722 mg/L for OD_660_ = 0.2, OD_660_ = 0.4, and OD_660_ = 0.6, respectively. This indicates that the *R. pal* exhibited a significant production of lycopene during electrochemical cultivation (*p* < 0.05).

### 3.4. Analysis and Comparison of Mutant Strains of R. pal

**Figure 4:**
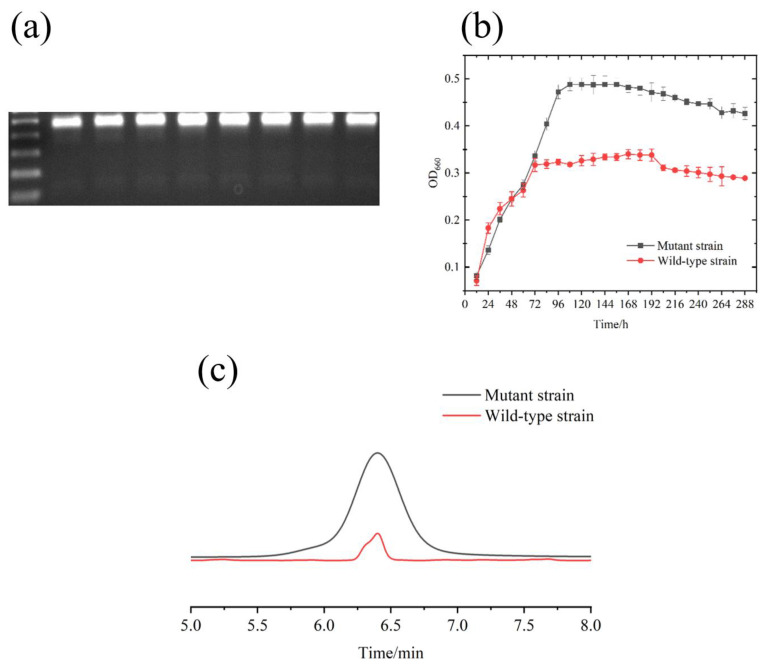
Comparison between original strain and mutant strain of *R. pal*. (**a**) Comparison of DNA gel electrophoresis between original strain and mutant strain. (lane 1: original strain at 24 h; lane 2: original strain at 48 h; lane 3: original strain at 72 h; lane 4: original strain at 96 h; lane 5: mutant strain at 24 h; lane 6: mutant strain at 48 h; lane 7: mutant strain at 72 h; lane 8: mutant strain at 96 h). (**b**) Comparison of electrochemical growth between original strain and mutant strain. (**c**) Liquid phase results of original strain and mutant strain of *R. pal*.

To further investigate the differences in electrochemical activity between the original strain and the mutant strain of *R. pal*, DNA was extracted from cultures of both strains at 24, 48, 72, and 96 h of cultivation. Gel electrophoresis was then performed on the extracted DNA samples. The experimental results indicate that DNA bands appeared around 1500 base pairs, and we observed that the brightness of the bands gradually increased (Figure 4a). It is noteworthy that the brightness of the lanes for the original strain was significantly lower than that of the mutant strain, indicating that the genes of the mutant strain underwent considerable changes under the electrochemical cultivation conditions.

To investigate whether the mutant strain exhibited better electrochemical activity compared to the original strain, both strains were subjected to electrochemical cultivation for a total of 12 days under identical conditions. The cultivation conditions for both strains were the same, and their OD_660_ values were measured every 12 h to compare their electrochemical characteristics. The results indicate that during the initial 24 h of the 12-day cultivation period, the original strain exhibited higher electrochemical sensitivity and grew faster compared to the mutant strain (Figure 4b). This may be attributed to the original strain being the first generation and possessing more active biological characteristics. However, as the cultivation time progressed, by 48 h, the growth patterns of the original strain and the mutant strain began to converge, and their growth curves began to approach each other. Subsequently, during the remaining cultivation period, we observed that the growth rate of the mutant strain gradually surpassed that of the original strain. Under electrochemical cultivation conditions, the mutant strain exhibited typical growth with higher OD_660_ values, reaching a maximum of 0.47, whereas the maximum growth density of the original strain was only 0.3.

Additionally, riboflavin is a product closely related to electron transfer on the microbial membrane. The removal of riboflavin from the microbial membrane can reduce electron transfer efficiency by 70%. Therefore, 500 μL of culture broth from the original and mutant strains with OD_660_ = 0.4 was taken, and riboflavin was extracted for quantitative analysis using HPLC. The experimental results are presented in Figure 4c. The experimental results show that the liquid-phase peak area of extracted menaquinone from the mutant strain of *R. pal* was 140.295 mAU, whereas that of the original strain was 66.5363 mAU. According to the linear regression equation, the menaquinone content extracted from the original strain of *R. pal* at OD_660_ = 0.4 was 5.229 mg/L, while the menaquinone content extracted from the mutant strain at OD_660_ = 0.4 during electrochemical cultivation was 61.081 mg/L. The statistical significance of the original and mutant strains was determined using an independent samples *t*-test. The menaquinone content extracted from the mutant strain after lysis was more than 10 times that of the original strain (*p* < 0.01). These results collectively indicate a substantial enhancement in the electrochemical activity of the mutant strain compared to the original strain.

### 3.5. Bioinformatic Analysis of the Mutant Strain

In order to clarify the enhanced electrochemical properties of the mutant strain even more, we performed gene function annotation and electrotolerance association analyses of the mutant and original strains by genome sequencing that revealed their differences at the gene level, providing important clues for the further exploration of the molecular mechanisms of electrotolerance. The mutant strain underwent whole-genome sequencing and assembly, resulting in a total sequence length of 6,029,600 bp, with a GC content of 61.82% and a Q30 percentage of 91.36%. The annotation of the genome using prokka software identified a total of 11,241 coding sequences and 218 non-coding sequences, including 106 tRNA sequences, one CRISPR sequence, 100 misc_RNA sequences, nine rRNA sequences, and two tmRNA sequences. As shown in Table 3, for the original strain, whole-genome sequencing and assembly yielded 5,095,116 bp sequences with a GC content of 62.73% and a Q30 percentage of 91.84%. The genome annotation revealed 9634 coding sequences, 99 misc_RNA sequences, nine rRNA sequences, 105 tRNA sequences, and two tmRBA sequences.

**Figure 5:**
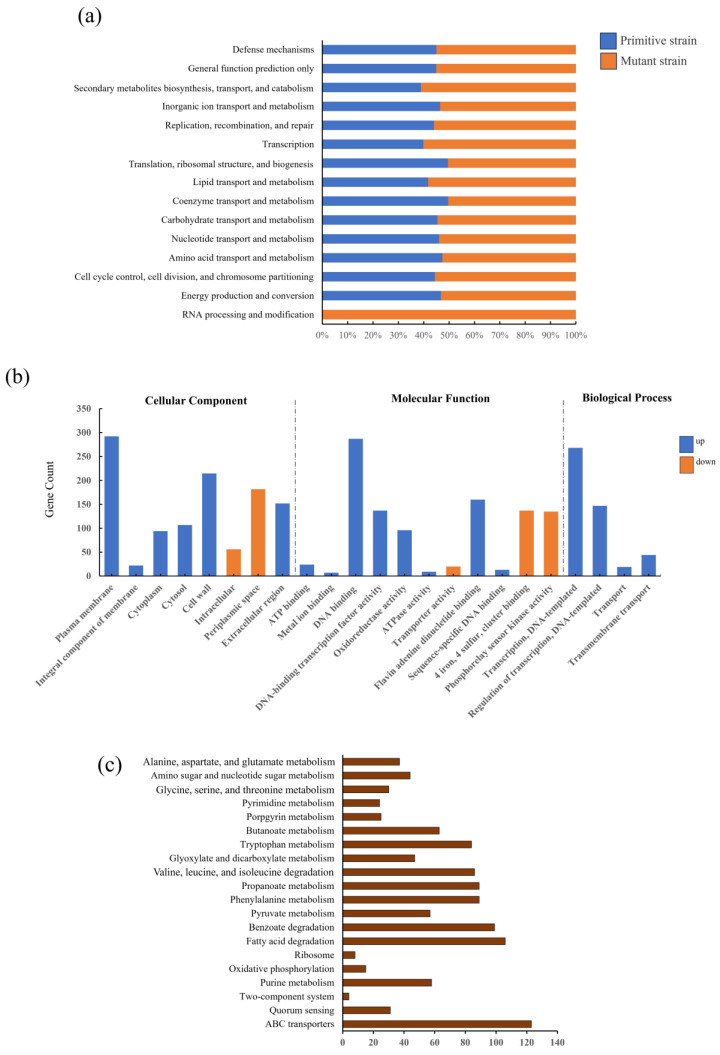
Comparison of genomes between original strain and mutant strain. (**a**) COG comparative analysis and (**b**) GO increase or decrease analysis. (**c**) Mutants grow on KEGG based on original strain.

Using blast software, the annotated gene sequences were compared with multiple databases. The original strain was annotated with 7542, 6463, 9498, 2935, 8284, 6823, 9572, and 9581 genes in the COG, GO, KEGG, GO, KOG, Pfam, Swissprot, and NR databases, respectively. In contrast, the mutant strain showed more annotated genes in these databases, with 8249, 7293, 10,934, 3382, 9410, 7744, and 11,136 genes annotated, totaling 11,153 genes. Further analysis revealed the enrichment of genes related to various pathways in the mutant strain compared to the original strain in the COG database. For instance, the mutant strain exhibited more genes enriched in pathways such as C, G, and I (Figure 5a).

In the GO database, the mutant strain primarily annotated genes related to the plasma membrane and integral components of the membrane in cellular components, with 2015 and 1424 genes, respectively. In molecular function, ATP binding and metal ion binding were the most enriched, with 1066 and 851 genes, respectively. In biological process, transcription, DNA templating, and the regulation of transcription had the highest enrichment, with 808 and 450 genes, respectively (Figure 5b). Additionally, in the KEGG database, the mutant strain-annotated genes were mainly enriched in pathways such as ABC transporters, the quorum sensing two-component system, purine metabolism, and oxidative phosphorylation (Figure 5c).

This study focused on riboflavin-related signaling pathways, comparing the mutant strain and the original strain in the GO and KEGG databases. The mutant strain annotated more genes related to riboflavin biosynthesis and metabolism compared to the original strain (Table 4).

### 3.6. Study on Riboflavin Pathway

**Figure 6:**
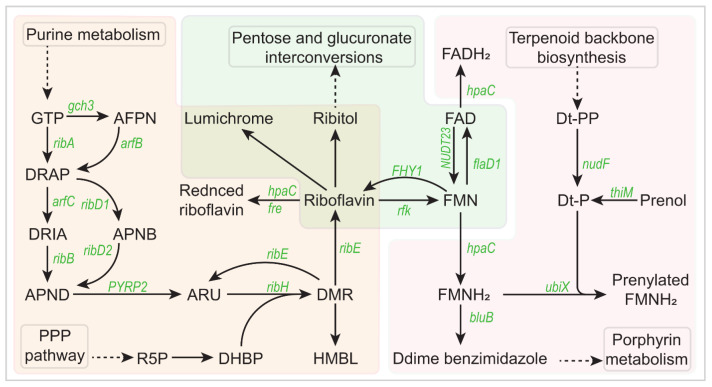
Riboflavin pathway analysis of mutant strain.

The synthesis of riboflavin is closely related to the electrochemical activity of *Azotobacter vinelandii*. The purine metabolism pathway provides GTP, which is one of the key substances for riboflavin synthesis. GTP undergoes enzymatic reactions to produce various metabolites, such as 2,5-Diamino6-(5-phosphe-L-ribosylamino) pyrimidin-4(3H)-one and 2-Amino-5-formylamino-6-(5-phospho-ribosylamino)-pyrimidin-4(3H)-one. Ribulose 5-phosphate, provided by the pentose phosphate pathway, is another precursor for riboflavin synthesis. Ribulose 5-phosphate undergoes specific enzyme reactions to ultimately yield 3,4-Dihydroxy-2-butanone 4-phosphate, a crucial intermediate in the riboflavin synthesis pathway. Riboflavin synthesis commences with 3,4-Dihydroxy-2-butanone 4-phosphate, which, through a series of enzyme-catalyzed reactions, gradually forms Riboflavin. Riboflavin further undergoes enzymatic modifications to yield various metabolites such as Lumichrome and Ribitol, which may participate in other biological metabolic pathways. Riboflavin can be converted into FMN (flavin mononucleotide) and FAD (flavin–adenine dinucleotide), both of which play crucial roles in cellular energy metabolism and redox reactions. FMN and FAD are interconvertible and participate in other metabolic pathways such as porphyrin metabolism. Through these pathways, purine metabolism and the pentose phosphate pathway provide the precursor substances required for riboflavin synthesis, while the riboflavin metabolism pathway is responsible for synthesizing riboflavin and its related metabolites, directing them towards other metabolic pathways to maintain metabolic balance and functional stability within the cell (Figure 6).

We observed differences in cellular components, molecular functions, and biological processes between the original and mutant strains as analyzed by the GO database. Specifically, the mutant strains had significantly more genes in the extracellular region than the original strains, while the original strains had slightly more genes in other cellular components (e.g., the cell, cell membrane, etc.) than the mutant strains. In terms of molecular function, the mutant strains possessed more genes for catalytic activity and binding function. In addition, the mutant strains were involved in a higher number and frequency of biological processes than the original strains, especially in metabolic processes, cellular processes, and processes in response to external stimuli. These differences may be related to the fact that the mutant strains had greater electrical tolerance. The mutant strains showed an advantage in electrical tolerance, which may have prompted adaptive changes at the genetic level in response to environmental stresses. The results showed that the mutant strains were superior in terms of the richness and diversity of gene functions, which may provide a genetic basis for their adaptability and competitiveness in different environments.

## 4. Conclusions

In this study, we investigated the application of *R. pal* within a dual-chamber microbial electrosynthesis system to facilitate the efficient production of lycopene. Through the study, it was found that the factors affecting the production of lycopene were mainly in the following aspects. (1) The characteristics of the strain itself: *Pseudomonas swampii*, as an experimental strain, has its own unique electrochemical activity and metabolic ability, which is the basis of being able to synthesize lycopene. For example, it can synthesize high-value-added compounds, including lycopene, from CO_2_ and waste glycerol using electrons under anaerobic conditions. (2) The culture’s environmental conditions and electrochemical conditions: The applied current and electron enrichment have important effects on lycopene production. In a two-cell electrosynthesis system, electron enrichment at the cathode provides the strain with electrons that promote lycopene synthesis. For example, *Pseudomonas swampii* at the cathode was able to grow and synthesize lycopene, while at the anode, it was not able to grow and synthesize lycopene due to different electron conditions. (3) Light conditions: *Pseudomonas aeruginosa* is a photosynthetic bacterium. In the experiment, the strain was in a light condition for a long period of time; although the light led to the oxidation of lycopene, it also showed that light is one of the factors affecting its growth and lycopene synthesis. However, this paper did not study the specific effects of different light intensities and durations on lycopene production. (4) Medium components: The components of the medium, such as various inorganic salts (0.5 M Na_2_HPO_4_, 0.5 M KH_2_PO_4_, 10% (NH_4_)_2_SO_4_, etc.) and added yeast powder in the PM medium, provided nutrients for the growth of the strain and the synthesis of lycopene. Lycopene synthesis provided the nutrient base. (5) Bacterial growth: The experimental results showed that lycopene production was linearly correlated with the concentration of bacterial cells. When the OD_660_ values of the bacteria were 0.2, 0.4, and 0.6, the corresponding lycopene production was different, and with the increase in the OD_660_ value, the lycopene production increased, which indicated that the growth state of the bacteria (cell concentration) affected the production of lycopene.

Through in-depth experimental analysis and bioinformatic assessments, several pivotal findings have emerged. Firstly, our results demonstrate the successful biosynthesis of lycopene by *R. pal* under electrochemical cultivation conditions. The lycopene concentration achieved 282.3722 mg/L, which is significantly higher than that obtained through traditional methods, thereby highlighting the potential of microbial electrosynthesis as a sustainable and efficient strategy for lycopene production. Secondly, the analysis of mutant strains revealed considerable genetic adaptations in response to electrogenic growth conditions. The mutant strain exhibited enriched gene frequencies in pathways associated with transcriptional regulation, signal transduction, and amino acid metabolism, indicating intricate genetic adaptations to electrogenic environments. Furthermore, bioinformatic analysis revealed significant differences in gene annotation between the mutant and original strains, with the mutant strain displaying enhanced enrichment in pathways such as riboflavin biosynthesis and metabolism. This indicates the potential advantages of the mutant strain in specific metabolic pathways that are crucial for electrogenic growth.

Overall, this study offers valuable insights into the genetic basis of electro-resistance genes and presents a novel strategy for the efficient and energy-conserving production of lycopene. The findings contribute to the advancement of our understanding of microbial electrosynthesis and its potential applications in sustainable biomanufacturing. Future research may direct attention toward elucidating the molecular mechanisms underlying genetic adaptations in mutant strains and optimizing microbial electrosynthesis systems to enhance the production of valuable compounds beyond lycopene. In the future, this technology can be applied to sewage treatment systems, using *R. pal* to degrade organic matter in sewage into energy products, so as to realize wastewater resource utilization and energy recovery.

## Figures and Tables

**Table 1:** Composition of PM medium.

PM	1000 mL PM (mL)
ddH_2_O	800
0.5 M Na_2_HPO_4_	25
0.5 M KH_2_PO_4_	25
10% (NH_4_)_2_SO_4_	10
Concentrated solution	1
0.1 M Na_2_S_2_O_3_·5H_2_O	1
2 mg/mL P-aminobenzoic acid	1
	Constant volume to 1000 mL

**Table 2:** Composition of PBS medium.

PBS	1000 mL PBS (mL)
KH_2_PO_4_	0.24 g
Na_2_HPO_4_	1.44 g
KCL	0.2 g
DdH_2_O	800 mL

**Table 3:** Data output statistics table.

Sample ID	Read Sum	Base Sum	GC (%)	Q20 (%)	Q30 (%)
Primitive strain	5,095,116	1,528,534,800	62.73	96.77	91.84
Mutant strain	6,029,600	1,808,880,000	61.82	96.46	91.36

**Table 4:** Table of gene counts in riboflavin-related pathways.

Database	Functional Pathways	Number of Genes	
		Primitive Strain	Mutant Strain
GO	Riboflavin biosynthetic process	14	21
KEGG	Riboflavin metabolism	11	17

## Data Availability

The original contributions presented in this study are included in the article. Further inquiries can be directed to the corresponding author.
